# LEVEL (Logical Explanations & Visualizations of Estimates in Linear mixed models): recommendations for reporting multilevel data and analyses

**DOI:** 10.1186/s12874-019-0876-8

**Published:** 2020-01-06

**Authors:** Maria Jose Monsalves, Ananta Shrikant Bangdiwala, Alex Thabane, Shrikant Ishver Bangdiwala

**Affiliations:** 1 Facultad de Medicina y Ciencia, Universidad San Sebastián, Santiago, Chile; 2 University of Minnesota, Minneapolis, MN USA; 3 McMaster University, Hamilton, ON Canada; 4 Population Health Research Institute, Hamilton, ON Canada; 5 Institute for Social and Health Sciences, University of South Africa, Johannesburg, South Africa; 6 Department of Health Research Methods, Evidence and Impact, Population Health Research Institute, McMaster University Faculty of Health Sciences, 237 Barton Street East, DBCVSRI Building, #C2-210, Hamilton, Ontario L8L 2X2 Canada

**Keywords:** Multilevel models, Reporting guidelines, Variance partition coefficients, Multilevel diagram

## Background

Researchers have been utilizing linear mixed models (LMMs) for different hierarchical study designs and under different names, which emphasizes the need for a standard in reporting such models [1, 2]. Mixed effects models, multilevel data, contextual analysis, hierarchical studies, longitudinal studies, panel data and repeated-measures designs are some of the different names used when referring to study designs and/or analytical tools for correlated data. In addition, there is usually no distinction made between having a data structure that is multilevel, and having a research question that requires a multilevel analysis. There are multiple excellent tutorials on multilevel analyses [3–5]. However, there is inconsistency in how the results of LMMs are reported in the literature [6]. Casals et al. conducted a systematic review of how various LMMs were reported in the medical literature, and found that important aspects were not reported in most cases [6].

As an example, a cohort study of children that selects a sample of schools, then selects students within schools, and conducts multiple measurements over time in the same students, would be a 3-level dataset: with school as the highest level (Level 3), student as a lower level (Level 2), and time-point as the lowest level (Level 1). Repeated measurements of a variable over time within a student are likely to be similar, i.e. positively correlated. Also, values of a variable measured on students of a particular school may be more similar to each other than to the values of the same variable measured on students from different schools, i.e. they are also likely to be positively correlated. These within-level correlations reduce the overall information in the data. Considering the correlations typically leads to larger estimates of variances and consequently lower power if sample sizes are not increased at the design stage. At the analysis stage, incorporating random effects into a regression model is one way to acknowledge the variation among upper-level units. Random intercepts and random slopes help to attribute the variation in values of the outcome variable to the relevant levels and independent variables.

A standardized checklist for the reporting of multilevel data and the presentation of linear mixed models will promote adequate reporting of correlated data analyses. In this manuscript, we propose LEVEL (Logical Explanations & Visualizations of Estimates in Linear mixed models), a systematic approach for the presentation of studies with correlated data from multilevel study designs, with an accompanying checklist for standardizing the reporting of results from linear mixed models. These models are quite complex, and the intention of this manuscript is not to be a statistical tutorial, but to mention aspects of the study design and analysis methods that we propose should be addressed in a publication. We present the basics of a linear mixed model simply to introduce the terminology and to help understand the proposed reporting recommendations.

## Methods

### The linear mixed model

Written as an equation, the ‘null’ (no covariate) linear mixed model for a 2-level hierarchical study is:

$$ {Y}_{ij}=\mu +{\tau}_i+{\varepsilon}_{ij}, $$

where *i* = 1, …,*m* indexes the number of upper-level units, *j* = 1, …, *n*_*i*_ indexes the number of base-level units in the *i*^th^ upper-level unit, *μ* denotes the overall mean of the dependent random variable *Y*, *τ*_*i*_ is the random intercept effect of the i^th^ upper-level unit, and *ε*_*ij*_ is the random error of the j^th^ lower-level unit in the i^th^ upper-level unit. We assume Normal distributions for the random effects, such that ${\tau}_i\sim N\left(0,{\sigma}_I^2\right)$ and ${\varepsilon}_{ij}\sim N\left(0,{\sigma}_E^2\right)$, where ${\sigma}_I^2$ is the component of variation due to variability among upper-level units, and ${\sigma}_E^2$ is the residual component of variation due to variability among lower-level units. We assume that these two random effects are independent of each other.

By acknowledging multiple sources of variability and then attributing the variation to the appropriate level, the multilevel model can more accurately and precisely estimate the effects of all variables included in the model [7]. Variance components are used to calculate the “intra-level” or intraclass correlation coefficient (ICC), a statistic that quantifies the degree to which data at the lower level are correlated. The ICC, also referred to as the variance partition coefficient (VPC), is calculated by the following proportion,

$$ ICC= VPC=\frac{\sigma_I^2}{\sigma_I^2+{\sigma}_E^2}, $$

which helps answer the question: of the total variation in the outcome variable, how much is accounted for by the variation among the upper-level units? As the term ICC is often mistaken for an estimate of a correlation coefficient, we will use the more appropriate term VPC.

A VPC close to 0 suggests that little to no variation in the outcome is attributable to variation among upper-level units, so most of the variation in the outcome is among the lower-level units and thus there is little correlation among them. On the other hand, a VPC close to 1 suggests that most of the variation in the outcome is attributable to variation among upper-level units, so little variation is to be found among the lower-level units; thus, there is high correlation among them. Calculating the VPC can help determine the presence of correlation at the lower level and the need to account for it in the analyses. Interpretation of the magnitude of the ICC/VPC is context dependent.

**Fig. 1:**
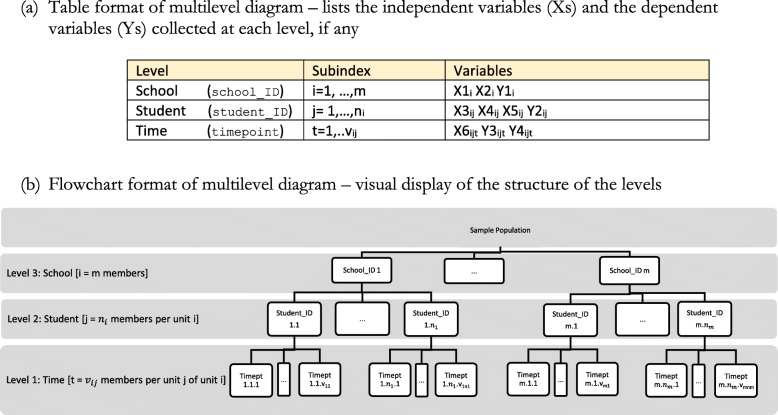
Schematic example of the ‘multilevel diagram’ for a 3-level hierarchical study of students nested in schools and repeated measurements over time in students, in **a** table format and **b** flowchart format

In hierarchical data structures with more than 2-levels (see *multilevel diagram* in Fig. 1), the VPC can be calculated for outcomes measured on units of each lower-level, with the numerator as the variation in outcome between units on all levels above [8]. For the example in Fig. 1, if we have the following ‘null’ model for the observation at time t on the j^th^ pupil from the i^th^ school,

$$ {Y}_{ij t}=\mu +{S}_i+{P}_{ij}+{\varepsilon}_{ij t}, $$

then VPC_1_ quantifies the correlation among all the values between and within pupils nested within schools and is given by

$$ {VPC}_1=\frac{\sigma_I^2}{\sigma_I^2+{\sigma}_J^2+{\sigma}_E^2}, $$

while VPC_2_ quantifies the correlation among the repeated measurements within pupils nested within schools and is given by

$$ {VPC}_2=\frac{\sigma_I^2+{\sigma}_J^2}{\sigma_I^2+{\sigma}_J^2+{\sigma}_E^2}, $$

where ${\sigma}_I^2$ is the component of variation due to variability among schools, ${\sigma}_J^2$ is the component of variation due to variability among pupils nested within schools, and ${\sigma}_E^2$ is the component of residual variation due to variability in the repeated measurements within pupils.

Understanding the implications that correlations among observations may have on the design and analyses of research studies is essential. At the design stage, if the contribution to the VPC for a particular level (the variance component) is small, it implies that there is little variation among units at that level; it is therefore more advantageous to sample more units from higher levels from an efficiency and power standpoint. These important statistical considerations in planning sample sizes at the different levels are accounted for with the variance inflation factor (VIF), also called the ‘design effect’. For a given level, k, the VIF is [1 + (m_k_-1) VPC_*k*_], where m_k_ is the average number of units in a member of the k^th^ level.

At the analysis stage, depending on the study design, linear mixed models can include random effects to account for correlation in space or in a social group (clustering), time (repeated-measures), or both. Table 1 presents example linear mixed models with dependent variable Y in hypothetical 2-level and 3-level study designs, with a single independent variable X. If the data were from a 1-level study design, the model would have no random effects (except the residual error!): *Y*_*j*_ = *β*_0_ + *β*_1_*X*_*j*_ + *ε*_*j*_, where ${\varepsilon}_j\sim N\left(0,{\sigma}_E^2\right)$.

**Table 1:** Example simple linear mixed models in 2-level and 3-level study designs

	Nature of design	Random intercept effects only	Random intercept effects and random slope effects
2-level	• Clustered	*Y*_*ij*_ = *β*_0_ + *β*_0*i*_ + *β*_1_*X*_*ij*_ + *ε*_*ij*_	*Y*_*ij*_ = *β*_0_ + *β*_0*i*_ + (*β*_1_ + *β*_1*i*_)*X*_*ij*_ + *ε*_*ij*_
	• No repeated measurements	${\beta}_{0i}\sim N\left(0,{\sigma}_I^2\right)$ ${\varepsilon}_{ij}\sim N\left(0,{\sigma}_E^2\right)$	${\beta}_{0i}\sim N\left(0,{\sigma}_{Iint.}^2\right)$ ${\beta}_{1i}\sim N\left(0,{\sigma}_{Islope}^2\right)$ ${\varepsilon}_{ij}\sim N\left(0,{\sigma}_E^2\right)$
2-level	• Not clustered	*Y*_*jt*_ = *β*_0_ + *β*_0*j*_ + *β*_1_*X*_*jt*_ + *ε*_*jt*_	*Y*_*jt*_ = *β*_0_ + *β*_0*j*_ + (*β*_1_ + *β*_1*j*_)*X*_*ij*_ + *ε*_*jt*_
	• Repeated measurements	${\beta}_{0j}\sim N\left(0,{\sigma}_J^2\right)$ ${\varepsilon}_{jt}\sim N\left(0,{\sigma}_E^2\right)$	${\beta}_{0j}\sim N\left(0,{\sigma}_{Jint.}^2\right)$ ${\beta}_{1j}\sim N\left(0,{\sigma}_{Jslope}^2\right)$ ${\varepsilon}_{jt}\sim N\left(0,{\sigma}_E^2\right)$
3-level	• Clustered	*Y*_*ijt*_ = *β*_0_ + *β*_0*i*_ + *β*_0*ij*_ + *β*_1_*X*_*ijt*_ + *ε*_*ijt*_	*Y*_*ijt*_ = *β*_0_ + *β*_0*i*_ + *β*_0*ij*_ + (*β*_1_ + *β*_1*i*_ + *β*_1*ij*_)*X*_*ijt*_ + *ε*_*ijt*_
	• Repeated measurements	${\beta}_{0i}\sim N\left(0,{\sigma}_I^2\right)$ ${\beta}_{0 ij}\sim N\left(0,{\sigma}_J^2\right)$ ${\varepsilon}_{ijt}\sim N\left(0,{\sigma}_E^2\right)$	${\beta}_{0i}\sim N\left(0,{\sigma}_{Iint.}^2\right)$ ${\beta}_{0 ij}\sim N\left(0,{\sigma}_{Jint.}^2\right)$ ${\beta}_{1i}\sim N\left(0,{\sigma}_{Islope}^2\right)$ ${\beta}_{1 ij}\sim N\left(0,{\sigma}_{Jslope}^2\right)$ ${\varepsilon}_{ijt}\sim N\left(0,{\sigma}_E^2\right)$

Note: Clusters are indexed by i, Subjects are indexed by j, and Time points are indexed by t

The random effects applied in the simple linear mixed models in Table 1 are assumed to have Normal distributions and to be independent from the error distribution. If there is more than one random effect, one must also specify if they are independent amongst themselves, and if not, should specify the covariance structure amongst the random effects.

The statistical literature is confusing and contradictory as to whether to consider effects as fixed or as random [9]. Many textbooks state that level effects must be considered as fixed effects if all possible members of that level were studied, and as random effects if members of that level are a sample from some population. Others state that fixed effects are to be used if the specific member effects are of interest, and as random effects if not. The Hausman test for the difference between the within-level and between-level regression coefficients is sometimes used as a test for deciding whether to use a random or fixed coefficient model [10]. We are not stating a position on this argument, but insist that one must acknowledge the hierarchical study design, not ignore the correlations, and justify the random intercepts and random slopes used.

### Multilevel data versus multilevel research question

The first step in analyzing multilevel data is to decide if the research question is a multilevel question. The design of a study may be hierarchical and thus have correlated data, but the research question may be one that does not require multilevel analyses. For example, in a clustered study design, research questions where the dependent variable is at the highest level will not require multilevel analyses since the members of the highest level are uncorrelated. In this case, the variation amongst members of the upper level is the only variance component, and a fixed effects model analyzed by ordinary least squares (OLS) is appropriate [10]. In a repeated measures *study design*, if the dependent variable in the research question is at a single time point, it is not a multilevel *question* as there are no repeated measures. Also, if the dependent variable is the time to the occurrence of an event (survival data), the research question is no longer multilevel; unless there is additional hierarchical structure, as in ‘frailty’ models. In a 2 or more level hierarchical clustered study, any research question using as dependent variable any lower level variable will require a multilevel analysis.

The next step is to consider using the multilevel diagram [8] as presented in Fig. 1. The multilevel diagram allows visualizing the levels of a study, the structure of the levels, and the variables collected at each level. Variables collected at higher levels than the dependent variable are usually called contextual variables. The diagram readily allows one to see if the dependent variable for a particular research question requires a multilevel analysis.

Another important consideration are ‘aggregated’ or ‘collapsed’ variables, which are variables derived by summarizing the values of observations from lower levels. For example, if years of education is available at the individual level for each adult in a household, the variable ‘highest education level of the household’ is an aggregated variable at the household level. If we have the sex and the grade-points for each student in multiple schools, the proportion of boys per school and the school-wide average grade-point are school-level aggregated variables.

Note that for a research question to be multilevel, the crucial decision is whether the dependent variable is at a lower level. One can have independent variables at a different (lower) level, but if the dependent variable is at the highest level, it is not a multilevel research question. For example, in a repeated measures design, the outcome at the end of treatment for a given person (e.g. treatment success) is measured only once, but may depend on values of a variable measured at different time points (e.g. hypertension at baseline and at times t1 and t2 prior to end of treatment).

## Results

### How to report descriptive analyses

With a hierarchical study design, a correct multilevel descriptive analysis should include analyses of the outcomes of interest at all relevant levels and distribution of the variables in all levels. This step will also help the researcher uncover irregularities in the data, such as unusual patterns of missingness, lack of heteroscedasticity, or unusual shapes of distributions. It is also helpful in understanding which variables are correlated and how to possibly consider them in the modeling.

The choice of summary statistics to use, as with non-multilevel descriptive statistical analysis, will depend on the type of variable. When presenting summary statistics (e.g. means for continuous variables, proportions for categorical variables) of variables collected at lower levels, measures of variability and confidence intervals must account for the variance inflation factors (VIFs).

When presenting plots, univariate and bivariate graphs should allow comparison of variables measured at the same level. With clustered data, plots of lower level variables should identify membership in upper level groups. With longitudinal data, plots of repeated measurements over time should identify points that come from the same subject (e.g. ‘spaghetti plots’) rather than summaries over time that obscure the fact that some of the same subjects are included across the summaries [11].

### How to report modeling analyses

Descriptive bivariate analyses that assess significance of correlation and association measures should adjust for the correlation in the observations. Once the focus shifts to the dependent variable of interest, the correlation among the observations of the dependent variable of interest at each level must be studied and presented. Variance decomposition must be performed and the VPCs or ICCs should be reported. An initial ‘null’ multilevel model with no independent variables is strongly encouraged.

The modeling, variable selection, and arriving at a ‘final’ model, is a process that every investigator can follow according to their choice, and is therefore not addressed. Note that adding dummy (indicator) variables as fixed effects for members of a higher level is not exactly equivalent to adding random intercept effects for members of a higher level. While both approaches do have the effect of explaining some of the variability in the outcome, only the latter decomposes the residual variance into components.

For the ‘final’ model, in addition to reporting the results for the fixed effects, one must report either the variance components or the VPCs or ICCs. It may be of special interest to report these for the ‘null’ model (i.e. with no independent variables), as well as for the final model (and other ‘intermediate’ models), so that the reader may understand the impact of explanatory variables on the variance components. Note also that if random intercepts and random slopes are included in the models, the estimated correlation structure among the random effects should also be presented. Finally, measures of model fit, such as either the Akaike Information Criterion (AIC) or the Bayesian Information Criterion (BIC), or the area under the receiver operating characteristic (ROC) curve (AUC) for logistic regression models, may be useful for readers.

### Example of reporting multilevel data structure and analyses: the Chilean dental study

We use a 3-level study that measured presence of caries in temporary dentition in 2275 children from 40 pre-schools in 13 districts (*comunas*) of the Metropolitan Region (around the capital of Santiago) in Chile, to illustrate what and how to present results from a multilevel analysis. All the districts in the Metropolitan Region were classified according to the United Nations Development Program (UNDP) Human Development Index (HDI) [12], then stratified into 5 groups: Very High, High, Middle, Low, and Very Low. Estimation of the necessary number of children and pre-schools to include took into account the expected ICCs and VIFs based on the literature. Thirteen districts were randomly selected across the strata. Within a district, educational establishments (pre-schools) were categorized into private (paid), private (subsidized) or public, and approached for participation. All selected districts participated, but the private pre-schools of the highest HDI district refused to participate; thus that district only had public (municipal) pre-schools participating. All eligible children of a school were invited to participate, and refusal (by parents) rates were less than 1%. The study was approved by the Comité de Ética de Investigación en Seres Humanos (ethics committee) of the Facultad de Medicina of the Universidad de Chile.

Table 2 displays the multilevel diagram for this study. The research question was: ‘Which factors are related to the presence of caries in temporary dentition in children of different districts of the Metropolitan Region?’ The prevalence of caries in temporary dentition in a group can be calculated from the presence of caries in temporary teeth at the individual-level. We note that our dependent variable is at a lower level, while the independent variables of interest are from various levels.

**Table 2:** Example multilevel diagram in table format for The Chilean Dental Study

Sub-index	Level	Variables
I (3)	District	HDI (Community-wide human development index) Rural location
J (18)	School	Administrative dependency of the school: private (paid), private (subsidized) or public School fluoride program: available or not
K (29)	Child	Age Sex Presence of gingivitis Educational level of main caretaker Family income Access to health care: private or public Frequency of tooth brushing: daily, 3–4 times/week, 2 times/week, once per week
		*Dependent variable:* Presence of caries in temporary teeth

Table 3 presents the results of three different random-intercept logistic regression models: the ‘null’ model, an ‘intermediate’ model, and a ‘final’ model, fitted using maximum likelihood. Usually only a final model is presented, but we illustrate how the other models can help in understanding changes in the VPC when one introduces independent variables from different levels in multilevel models. See model equations in the Additional file 1.

**Table 3:** Random-intercept logistic regression models for the presence of caries in The Chilean Dental Study

	Value of category	‘Null’ model (*n* = 2275)	‘Intermediate’ model (*n* = 2275)	‘Final’ model (*n* = 2134)^b^
		OR (95% CI)	OR (95% CI)	OR (95% CI)
District-level variables				
Human Development Index			0.08 (0.01–0.82)	0.04 (0.01–0.39)
Rural location			1.82 (1.17–2.81)	1.45 (0.98–2.16)
School-level variables				
Administrative dependency	Private (paid)		*Reference*	*Reference*
	Private (subsidized)		2.74 (1.64–4.55)	1.12 (0.69–1.83)
	Public		3.99 (2.28–6.98)	1.65 (0.98–2.77)
School fluoride program	No program		1.33 (0.84–2.12)	1.17 (0.81–1.70)
Child-level variables				
Sex	Male			1.22 (1.00–1.48)
Age	1 year			*Reference*
	2 years			4.98 (2.18–11.38)
	3 years			12.14 (5.45–27.04)
	4 years			15.00 (6.69–33.64)
	5 years			15.22 (6.68–34.68)
	6 years			14.34 (6.20–33.23)
Family income				0.93 (0.85–1.02)
Presence of gingivitis	Absent			*Reference*
	Present			2.14 (1.67–2.75)
Educational level of main caretaker	University			*Reference*
	No studies			0.90 (0.14–5.73)
	Primary school			1.26 (0.81–1.97)
	Secondary school			1.60 (1.14–2.24)
	Technical school			1.15 (0.82–1.61)
Access to health care	Private			*Reference*
	Public			1.21 (0.89–1.65)
ICC^a^ district		0.0495	0.0135	0.0134
ICC^a^ school within district		0.1278	0.0378	0.0153
AIC^a^		2863.3	2836.8	2521.6
AUC^a^		0.50 (0.50–0.50)	0.63 (0.60–0.65)	0.72 (0.70–0.74)

^a^*ICC* intra-level correlation coefficient, *AIC* Akaike Information Criterion, *AUC* area under the ROC curve

^b^Note that the number of children in this model is lower due to non-response to various variables; ‘family income’ had the highest number (76) of non-responders (3.3%)

The effect estimates and 95% confidence intervals (CIs) do account for the correlation among the observations; at the bottom of the table of results, one presents the corresponding intraclass correlation coefficients and the model fit criteria.

We first note that in the intermediate model, which only includes district-level and school-level covariates, the district-level variables of HDI and rurality, and the type of school are statistically significant – the higher the human development index of the district, the lower the probability of caries among the children, while children in private (paid) pre-schools have lower probability of caries. In the final model, which now includes child-level covariates, the odds ratios (ORs) for school type and rural location are no longer significant. The sex and age of the child are significant, while family income and access to health care were not significantly associated with caries presence. Secondary school education of the main caretaker was associated with higher likelihood of caries. It could be that district-level factors like HDI account for the effect of child-level socioeconomic factors.

From the ‘null’ model, we note that the correlation of the presence of caries of children from the same district is not negligible (ICC = 0.0495), but also that this correlation is more than doubled (ICC = 0.1278) among children within the same school. When we consider district-level and school-level covariates, the ICC for district and for school within district are reduced. The ICC for district is not reduced further when we add child-level covariates in the ‘final’ model. However, the correlation among presence of caries among children within the same school is reduced when child-level covariates are included in the model.

The final model, as expected, has a much better fit than the intermediate model (much lower AIC), since it incorporates child-level covariates, which explain well the child-level variable of presence of caries.

## Discussion

The objective of this manuscript is to recommend how to report and present multilevel data and the results of linear mixed models. The need for such a checklist has been previously established by Casals et al. [6], who conducted a systematic review of the quality of the presentation of results and information from LMMs in the field of clinical medicine. Their extensive and systematic review of indexed medical journals included longitudinal studies, repeated measurements and multilevel design studies, from various medical disciplines. They found that “most of the useful information about generalized linear mixed models was not reported in most cases.” [6] Less than 10% reported the variance estimates of random effects. Aspects that apply to all modeling, such as covariate selection, estimation method, and goodness of fit, were also not universally reported. They conclude that “it is important to consider the use of minimal rules as standardized guidelines when presenting generalized linear mixed model results in medical journals.” [6]

This manuscript is limited since it is not intended to be a tutorial on statistical methods for analyzing correlated data. Many such tutorials do exist. We do not review the complex statistical considerations behind all the aspects that are important in LMMs. We provided a real-data example using a mixed effects logistic regression analysis of a 3-level study to illustrate how they such analyzes could be reported following our recommendations.

Table 4 presents a checklist of items that we recommend for reporting multilevel data and modelling results, where items are either suggested (S), expected (E) or necessary (N). The checklist was developed by the authors based on their experience in conducting and presenting multilevel data analyses. We thus welcome comments from users of the proposed checklist and from journal editors. We welcome considering extending our recommended checklist to other multilevel models. Checklists such as the PRISMA [13], STROBE [14], CONSORT [15] and others have improved the quality of reporting of scientific medical research studies in abstracts and full manuscripts [16]. More recently, reporting guidelines for models have been proposed [17, 18]. The proposed LEVEL checklist is modeled on STROBE guidelines, modified for multilevel studies.

**Table 4:** LEVEL (Logical Explanations & Visualizations of Estimates in Linear mixed models) checklist of items for reports of multilevel data and modelling analyses

	Item	Recommendation
Title and abstract	1	(*a*) Not essential in the title, but the fact that the study is hierarchical and the analyses are multilevel must be mentioned in the abstract (S)
		(*b*) The abstract should mention the various levels considered in the analyses and whether random intercepts only or also random slopes were modeled (N)
Introduction		
Background/rationale	2	Provide rationale for the study design being hierarchical and for the analyses being multilevel (E)
Objectives	3	Mention at what level the dependent and independent variables are taken (S)
Methods		
Study design	4	(*a*) Provide the multilevel diagram for the study (S)
		(*b*) Justify level of the analyses (N)
Population	5	(*a*) Provide the number of members of each level, the eligibility criteria, and the sources and methods of selection/sampling of the members (N)
		(*b*) If a repeated measures design, provide description of methods of follow-up, and spacing of time points (N)
		*(c)* Describe missingness patterns and imbalances in members across levels (E)
Variables/ data structure	6	(*a*) Write out the multilevel model equation including the random effects – this may be provided in an Appendix (N)
		(*b*) Mention the variables used and from what level (N)
Study size	7	(*a*) Provide details of the sample size calculation, and mention relevant variance partition coefficients (VPC) or intraclass correlation coefficients (ICC) and variance inflation factors (VIF) for each level (N)
		(*b*) Provide justification for ICCs from previous studies – literature or pilot studies (E)
Statistical methods	8	*(a)* Describe all statistical methods, descriptive and inferential, detailing how the correlation in the data was dealt with (E)
		*(b)* Mention estimation procedure utilized (e.g. restricted maximum likelihood) (S)
		*(c)* Present variance components or VPCs/ICCs for ‘null’ model and for final model (S)
		*(d)* Justify variables considered in the initial model and justify the ones included in the final model (N)
		*(e)* Justify choice of random or fixed intercepts and random or fixed slopes for variables in the final model, along with correlation structure among the random effects (N)
Results		
Participants	9	*(a)* Report the number of individuals from each level in the final model, since missing data may affect the original numbers (N)
		*(b)* Present a flow diagram (S)
Descriptive data	10	*(a)* Indicate number of participants with missing data for each variable of interest, by level (S)
		*(b)* Identify the level when presenting graphs and tables (E)
		*(c)* Adjust the variances even in descriptive univariate or bivariate analyses (N)
Modeling results	11	*(a)* Present the model equation and estimates – maybe in Appendix (S)
		*(b)* Present a summary table with estimates of fixed effects, VPCs/ICCs for null model, intermediate models (if any) and final model (N)
		*(c)* Present model goodness of fit statistics (N)
Other analyses	12	Report other analyses and if multilevel, provide similar information as above (S)

Key: *S* Suggested, *E* Expected, *N* Necessary

## Conclusions

A standardized checklist for the reporting of multilevel data and the presentation of linear mixed models will promote adequate reporting of correlated data analyses, and ensure that appropriate statistics are contained and explained thoroughly in manuscripts. The implementation of our checklist of items to report when presenting results of a multilevel analysis hopes to increase transparency, completeness, and the quality of reporting.

## Supplementary information

**Additional file 1.** Model equations for the Example mixed effects logistic regression models used for The Chilean Dental Study. Three model equations are provided: 1. ‘Null’ logistic regression model – no independent variables. 2. ‘Intermediate’ logistic regression model – with selected district- and school-level independent variables. 3. ‘Final’ logistic regression model – with selected district, school- and child-level independent variables.

## Data Availability

The datasets used and/or analysed during the current study are available from the first author (maria.monsalves@uss.cl) on reasonable request.

## Abbreviations

AIC: Akaike Information Criterion
AUC: Area under the receiver operating characteristic curve
BIC: Bayesian Information Criterion
CI: Confidence interval
CONSORT: CONsolidated Standards Of Reporting Trials
E: Expected
HDI: Human Development Index
ICC: Intraclass correlation coefficient
LEVEL: Logical Explanations & Visualizations of Estimates in Linear mixed models
LMM: Linear mixed model
N: Necessary
OLS: Ordinary least squares
OR: Odds ratio
PRISMA: Preferred Reporting Items for Systematic reviews and Meta-Analyses
ROC: Receiver operating characteristic
S: Suggested
STROBE: Strengthening The Reporting of OBservational studies in Epidemiology
UNDP: United Nations Development Program
VIF: Variance inflation factor
VPC: Variance partition coefficient
