# Supplementary material for: LEVEL (Logical Explanations & Visualizations of Estimates in Linear mixed models): recommendations for reporting multilevel data and analyses
Source: BMC Med Res Methodol. 2020 Jan 6;20:3. doi: 10.1186/s12874-019-0876-8 (PMC6945753; doi:10.1186/s12874-019-0876-8)
Supplement: Supplementary file 1 — Additional file 1. Model equations for the Example mixed effects logistic regression models used for The Chilean Dental Study. Three model equations are provided: 1. ‘Null’ logistic regression model – no independent variables. 2. ‘Intermediate’ logistic regression model – with selected district- and school-level independent variables. 3. ‘Final’ logistic regression model – with selected district, school- and child-level independent variables. [file 12874_2019_876_MOESM1_ESM.docx]

**Model equations for the Example mixed effects logistic regression models used for**

**The Chilean Dental Study**

The mixed effects logistic regression model is used for correlated binary outcome variables. In it, the log odds (logit) of the probability of the binary outcome is modeled as a linear combination of the predictor variables. The ICC is estimated by assuming that ‘latent errors’ are logistic with mean 0 and variance

1. ‘Null’ logistic regression model – no independent variables:

$${logit Pr(Presence of caries}_{ijk}=yes)=\beta_{0}+\beta_{\begin{aligned} 0i \\ \end{aligned}}+\beta_{0ij}$$

2. ‘Intermediate’ logistic regression model – with selected district- and school-level independent variables:

$${logit Pr(Presence of caries}_{ijk}=yes\left| selected variables \right)=\beta_{0}+{\beta_{\begin{aligned} 0i \\ \end{aligned}}+ \beta_{0ij}+\beta}_{1}\mathrm{HDI}_{i}{+\beta}_{2}{rural location}_{i}{+\beta}_{3}{Administrative dependency of the school}_{ij}{+\beta}_{4}{School fluoride program}_{ij}$$

3. ‘Final’ logistic regression model – with selected district, school- and child-level independent variables:

$${logit Pr(Presence of caries}_{ijk}=yes|selected variables)=\beta_{0}+{\beta_{\begin{aligned} 0i \\ \end{aligned}}+ \beta_{0ij}+\beta}_{1}\mathrm{HDI}_{i}{+\beta}_{2}{rural location}_{i}{+\beta}_{3}{Administrative dependency of the school}_{ij}{+\beta}_{4}{School fluoride program}_{ij}+\beta_{5}\mathrm{Age}_{ijk}+\beta_{6}\mathrm{Sex}{}_{ijk}+\beta_{7}\mathrm{Gingivitis}_{ijk}+\beta_{8}{Educational level}_{ijk} +\beta_{9}{Family income}_{ijk} +\beta_{10}{Health care access}_{ijk}$$
